# Thermal and Mechanical Properties of Biocomposites Made of Poly(3-hydroxybutyrate-*co*-3-hydroxyvalerate) and Potato Pulp Powder

**DOI:** 10.3390/polym11020308

**Published:** 2019-02-12

**Authors:** Maria Cristina Righetti, Patrizia Cinelli, Norma Mallegni, Andreas Stäbler, Andrea Lazzeri

**Affiliations:** 1 CNR-IPCF, National Research Council—Institute for Chemical and Physical Processes, Via Moruzzi 1, 56124 Pisa, Italy; norma.mallegni@pi.ipcf.cnr.it (N.M.); andrea.lazzeri@unipi.it (A.L.); 2 Department of Civil and Industrial Engineering, University of Pisa, Largo Lucio Lazzarino 1, 56122 Pisa, Italy; 3 Fraunhofer Institute for Process Engineering and Packaging IVV, Giggenhauser Straße, 35, 85354 Freising, Germany; andreas.staebler@ivv.fraunhofer.de

**Keywords:** bio-based polymers, natural fibers, biomass, biocomposites, fiber/matrix adhesion

## Abstract

The thermal and mechanical properties of biocomposites of poly(3-hydroxybutyrate-*co*-3-hydroxyvalerate) (PHBV) containing 5 wt % of valerate units, with 20 wt % of potato pulp powder were investigated in order (*i*) to obtain information on possible miscibility/compatibility between the biopolymers and the potato pulp, and (*ii*) to quantify how the addition of this filler modifies the properties of the polymeric material. The potato pulp powder utilized is a residue of processing for the production and extraction of starch. The final aim of this study is the preparation of PHBV based materials with reduced cost, thanks to biomass valorization, in agreement with the circular economy policy, as result of the incorporation of agricultural organic waste. The results showed that the potato pulp powder does not act as reinforcement, but rather as filler for the PHBV polymeric matrix. A moderate loss in mechanical properties is detected (decrease in elastic modulus, tensile strength and elongation at break), which regardless still meets the technical requirements indicated for rigid packaging production. In order to improve the mechanical response of the PHBV/potato pulp powder biocomposites, surface treatment of the potato pulp powder with bio-based and petroleum-based waxes was investigated. Good enhancement of the mechanical properties was achieved with the natural carnauba and bee waxes.

## 1. Introduction

Biodegradable bio-based polymers, obtained from renewable resources, represent an important alternative to petrol-derived non-degradable polymers. Thus bio-based polymers have become an important issue both for academia and industry. Typical examples are poly(lactic acid) (PLA), and polyhydroxylalkanoates (PHA), in particular polyhydroxylbutyrate (PHB) and its copolymers poly(hydroxylbutyrate-*co*-valerate) (PHBV).

Polyhydroxyalkanoates are a wide family of polyesters, obtained by different bacteria cultivated under stressful conditions, with properties quite similar to conventional plastics [1,2,3]. The commercially marketed PHBV copolymers have good mechanical properties [4] and good resistance to solubility in water [5]. PHB and PHBV are also highly biodegradable and biocompatible [6,7,8]. Unfortunately, the relatively high cost [3], compared to other biodegradable polymers such as PLA, has somehow hindered research activity on the use of PHB and PHBV in commodity applications such as packaging and service items, and restricted their use to high-value applications, such as those in medical and pharmaceutical sectors. 

In order to achieve products with particular properties for different applications, biocomposites can be utilized. Biocomposites are a special class of composite materials, obtained by mixing natural fibers to bio-based polymers. Biocomposites represent an environmentally friendly and low-cost alternative to conventional petroleum-derived materials. Their properties have been reviewed in several books and articles [9,10,11,12,13,14,15,16,17]. The additional benefit offered by natural or bio-based fibers is that, besides being biodegradable, they also exhibit a lower density, which makes biocomposites economical and lightweight [18].

The mechanical performances of a fibers reinforced biocomposite result from both the matrix and the fiber properties [19,20], and are strongly dependent on the fiber/matrix interphase [21]. The tensile strength is more sensitive to the matrix/fiber adhesion, whereas the modulus depends in general on both the matrix and the fiber properties. The percentage of fibers, their aspect ratio (length to width ratio) and orientation, and the fiber–matrix adhesion are crucial elements responsible for the final properties of natural fiber reinforced composites. The transmission of the applied stress to the fibers occurs at the interface, which explains the necessity of a good matrix/fiber adhesion. Often biocomposites made of hydrophobic polymers and hydrophilic natural fibers are characterized by poor adhesion, which results in limited mechanical properties, due to the tendency of the fibers to aggregate during processing. In addition, the hydrophilic nature of the lignocellulosic fibers usually causes moisture absorption, which worsens processability and induces formation of porous products. The fiber aspect ratio strongly influences the tensile modulus and the fracture properties. Fibers with low aspect ratio and irregular shape in general behave as fillers, and not as reinforcement.

The poor compatibility between fiber and biopolymeric matrix can be improved by modifying the fiber surface properties [17]. Physical and chemical methods can be utilized. Some physical treatments, as for example stretching, can enhance the interface polymer/natural fibers, without changing the chemical composition of the fibers. Plasma or corona treatment can induce compatibilization between hydrophilic fibers and hydrophobic matrix, through formation of free radicals and surface cross-linking. On the other hand, chemical methods utilize coupling agents to modify the surface composition of the fibers, or chemical treatments to increase the surface roughness or to reduce the fiber hydrophilic nature. 

Several types of natural fibers have been used to produce PHBV based biocomposites, for which thermal and mechanical properties were investigated [8,22,23,24,25,26,27]. Organic wastes have also been sometimes utilized as reinforcement or additives for different polymers [28]. Significant amounts of organic wastes from industry and agriculture remain unutilized, so that the use of organic residue materials in biocomposites can represent a sustainable method to produce materials for different applications, characterized also by reduced cost, meeting even a circular economy approach.

Potato wastes are biomasses rich in starch and lignocellulosic constituents. After extraction of starch, the potato pulp accumulates in high amounts—approximately 0.75 tons of pulp arises per ton of purified starch. Within the European Union, about 140,000 tons of dried potato pulps are produced annually in the starch industry [29]. The original potato pulp contains water up to about 90%, but de-watering processes generally results in an increase in the dry matter up to about 90 wt %. Dried potato pulp, which consists mainly of lignocellulosic fibers, starch and, at a lesser extent, of proteins, can be used as filler for reinforcement of biopolymers. As the starch content that remains after potato processing can be quite high, similar to that of the lignocellulosic fibers, the potato pulp powder can be defined as a mixture of lignocellulosic fibers and starch, both with hydrophilic structure. The cost of the potato pulp powder is low, which makes it even more interesting for industrial utilization [30]. Potato pulp powder has never been utilized to produce biocomposites with PHBV. In the present work, biocomposites made of PHBV and 20 wt % of potato pulp powder have been produced by extrusion, followed by injection molding, and characterized in terms of thermal, mechanical and morphological properties. Preliminary tests indicated that potato pulp powder could be added and easily processed with PHBV up to a percentage of about 20 wt %. Due to the high cost of PHBV, in order to investigate the cheapest formulation, in the present study the properties of the PHBV based biocomposite with 20 wt % of potato pulp powder were analyzed. To make the preparation of the biocomposite simpler, a plasticizer, acetyl-tri-n-butyl citrate (ATBC) was used. ATBC is in general an efficient plasticizer of PHAs [31]. It is derived from naturally occurring citric acid, is non-toxic and accepted for contact with food [32,33]. In addition, in an attempt to improve the adhesion between PHBV and potato pulp and reduce the tensions at the interface, in the present study fiber coating with bio-based and petroleum-based waxes was employed and investigated.

## 2. Materials and Methods

### 2.1. Materials

Commercial grade polyhydroxyalkanoate (PHI002™) was supplied in pellets by Naturplast^®^ (Caen, France). The material is a copolymer poly(3-hydroxybutyrate-*co*-3-hydroxyvalerate) (PHBV) with 5 wt % valerate content, characterized by a density of 1.25 g/cm^3^ and a melt flow index (190 °C, 2.16 kg) of 10–20 g/10 min.

The plasticizer acetyl-tri-n-butyl citrate (ATBC) was purchased from SigmaAldrich S.R.L. (Milan, Italy). 

The calcium carbonate (CaCO_3_) OMYACARB^®^ was an inert filler supplied by the company OMYA (Oftringen, Switzerland). The powder, which has fine granulometry with particle size distribution centered at 12 μm, is used to facilitate the removal of the injection molded specimen from the mold. 

The dried potato pulp (PP) powder was provided by the company (SüdStärke, Schrobenhausen, Germany). The moisture content was about 3 wt %, and the composition of the dry matter: cellulose 16 wt %, hemicellulose 7 wt %, lignin 20 wt %, starch 25 wt %, pectin 17 wt %, proteins 7 wt %, ash 5 wt %. 

Wax-based additives Aquacer 561, Aquacer 581, Aquacer 593 and Hordamer PE 02 were provided by BYK Additives & Instruments (Wesel, Germany). They are (*i*) non-ionic aqueous emulsions of bee wax (Aquacer 591), (*ii*) non-ionic aqueous emulsions of carnauba wax (Aquacer 581), (*iii*) non-ionic aqueous emulsion of a modified polypropylene wax (Aquacer 593), and (*iv*) anionic aqueous emulsion of polyethylene (Hordamer PE 02).

### 2.2. Composite Preparation

Biocomposites of PHBV with potato pulp powder were prepared by adding 20 wt % of PP powder to a polymeric matrix, constituted by the biopolymer PHBV, with concentration 85 wt %, the plasticizer ATBC, with concentration 10 wt %, and CaCO_3_, with concentration 5 wt %. For comparison, pure PHBV and the PHBV mixed only with the plasticizer ATBC were also processed in the same way. For the preparation of the PHBV based biocomposites with potato pulp powder coated with natural waxes, 20 mL of wax aqueous emulsion with concentration 5% *w*/*v* were added to 19 g of PP powder. The mixture was carefully hand blended for sufficient long time. The PHBV based samples investigated in the present study, with the relative composition, are listed in Table 1.

Before processing, PHBV and the PP powder, non-coated and coated, were dried at a temperature of 60 °C for at least 24 h. The PHBV based matrix and biocomposites were prepared by using a MiniLab II HAAKE Rheomex CTW 5, a co-rotating conical twin-screw extruder, which allows the mixing of the different components. The molten materials were transferred from the mini extruder through a preheated cylinder to a mini injection molder (Thermo Scientific HAAKE MiniJet II), which allows us to prepare dog-bone tensile bars specimens to be used for thermal and mechanical characterization. The dimensions of the dog-bone tensile bars were: width in the larger section: 10 mm, width in the narrow section: 4.8 mm, thickness 1.35 mm, length 90 mm. The extruder operating conditions adopted for all the formulations are reported in Table 2. After preparation, all the samples were stored in a desiccator and analyzed the day after in order to avoid physical ageing effects on the physical properties investigated.

### 2.3. Composite Characterization

The thermal stability of the potato pulp powder and selected samples was investigated by thermogravimetric analysis (TGA), carried out on about 10 mg of sample by using a Perkin Elmer TGA 7 (Waltham, MA, USA), under nitrogen flow (35 mL/min), at a heating speed of 10 K/min from 50 °C to 600 °C. 

The morphology of the potato pulp powder and PHBV based matrix and biocomposites was investigated by scanning electron microscopy (SEM) with an FEG-Quanta 450 ESEM instrument (Waltham, MA, USA). The micrographs of samples fractured with liquid nitrogen and etched with gold were collected. Backscattered electrons generated the images whose resolution was provided by beam deceleration with a landing energy of 2 kV.

Differential scanning calorimetry (DSC) measurements were performed with a Perkin Elmer Calorimeter DSC 8500 (Waltham, MA, USA) equipped with an IntraCooler III as a refrigerating system. The instrument was calibrated in temperature with high purity standards (indium, naphthalene, cyclohexane) according to the procedure for standard DSC [34]. Energy calibration was performed with indium. Dry nitrogen was used as purge gas at a rate of 30 mL/min. The samples were analyzed from −85 °C to 200 °C at the heating rate of 10 K/min.

Tensile tests on the samples prepared with the injection molder were performed at room temperature, at a crosshead speed of 10 mm/min, by means of an INSTRON 5500 R universal testing machine (Canton, MA, USA), equipped with a 10kN load cell and interfaced with a computer running the Testworks 4.0 software (MTS Systems Corporation, Eden Prairie, MN, USA). At least five specimens were tested for each sample in according to the ASTM D 638, and the average values reported.

## 3. Results and Discussion

### 3.1. Thermogravimetric Analysis of the Potato Pulp Powder, the PHBV Based Matrix and Biocomposites [PHBV(85 wt %)+ATBC(10 wt %)+CaCO_3_(5 wt %)](80 wt %)+PP(20 wt %)

**Figure 1:**
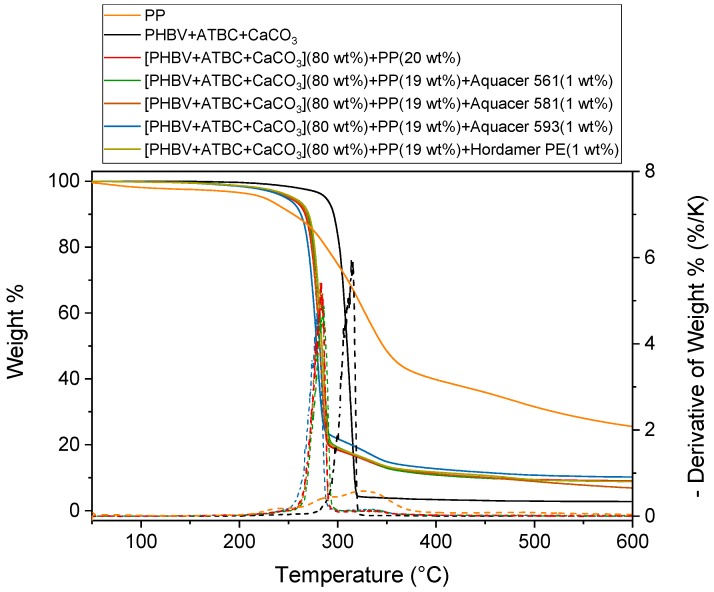
Thermogravimetric curves of the potato pulp powder (PP), the PHBV based matrix and biocomposite [PHBV(85 wt %) + ATBC(10 wt %) + CaCO_3_(5 wt %)](80 wt %) + PP(20 wt %) without and with wax treatment at 10 K/min under nitrogen flow (estimate error: ± 0.2 weight %). The dotted lines are the derivative of the weight % curves.

The thermal stability of the potato pulp PP was determined by means of the thermogravimetric analysis under nitrogen flow, because the contact of the material with air is reduced in the extruder and molder. Figure 1 shows the thermogravimetric curve of the PP powder, which reports the change in weight according to a fixed temperature program. Due to thermal degradation of the fiber components that occurs at high temperatures [35,36], a relatively low processing temperature is required to process biocomposites containing natural fibers. 

The initial weight loss, detected in Figure 1 at temperatures lower than 150 °C, is due to water vaporization. The water content of PP is approximately 3 wt %. The weight residue that is observed at high temperature is due to the carbon deposit that remains in the presence of an inert atmosphere. Thermal degradation of PP takes place in the temperature range 200°C–600 °C, and is due to degradation of hemicellulose, which occurs mainly in the range 200°C–350 °C, cellulose, which is generally observed between 250 °C and 400 °C, and lignin, which starts at about 250 °C [35,36]. Starch degradation extends from approximately 300 °C to 350 °C [37], and proteins degradation from 200 °C to 400 °C [38]. 

From Figure 1 the potato pulp powder appears stable up to approximately 190 °C. This proven thermal stability assures us that PP powder does not undergo substantial degradation during the processing of the PHBV biocomposites at 180 °C, with the residence time at this temperature being not longer than 1.5 min. 

Figure 1 shows also the the thermogravimetric curves of the PHBV based matrix and biocomposite with 20 wt % of PP without and with surface treatment of the potato pulp powder with waxes. The thermal degradations of the PHBV based matrix occurs in a single step in a narrow temperature range. The initial degradation temperature is located at about 300 °C, whereas the maximum degradation rate is centred at about 315 °C, in agreement with previous studies [39,40]. The thermal degradation of the biocomposites takes place in multiple steps, as cumulative process of the matrix and the filler. Independently of the wax treatment, biocomposites start to degrade at about 190 °C, whereas the maximum degradation rate shifts to around 280 °C. The temperature reduction of the main degradation step with respect to the PHBV matrix could be ascribed to a combined degradation process of the components, in the presence of the PP moisture [41]. As expected, the residue of the biocomposite at 600 °C is higher with respect to that of the PHBV based matrix, because of the lignocellulosic and starch residues. The thermogravimetric curves also show that potato pulp powder is the last component to undergo degradation. 

In conclusion Figure 1 reveals that the processing of the biocomposites at 180 °C does not substantially affects the potato pulp powder structure, because the degradation of the biocomposite starts at the same temperature as the original unprocessed potato pulp powder. It also shows that PP powder can be used for the production of PHBV based biocomposites.

### 3.2. Scanning Electron Microscopy of the Potato Pulp Powder

**Figure 2:**
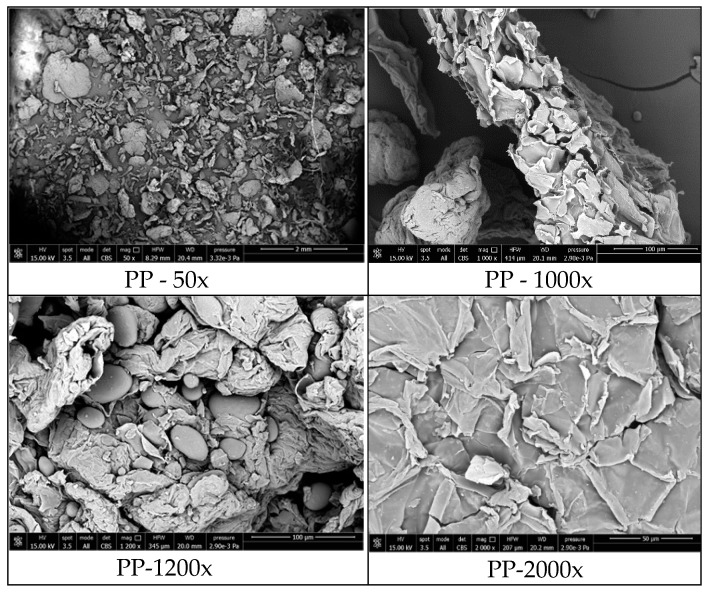
SEM images of the PP powder at the magnifications indicated.

Morphology of the potato pulp powder were investigated with scanning electron microscopy (SEM). Figure 2 reports the relative SEM images. A quite homogeneous distribution of the pulp fragments, which appear as small aggregates, can be observed. The aggregates are relatively large (200 µm and more). The round shaped particles detected at 1200× magnification in the PP powder are either starch or pectin, because they disappear after treatment with amylase and pectinase. 

### 3.3. Thermal, Mechanical and Viscoelastic Properties of the PHBV Matrix

The thermal and mechanical properties of the PHBV matrix were investigated as preliminary step, in order to better quantify the influence of the fibers on the material properties. 

**Figure 3:**
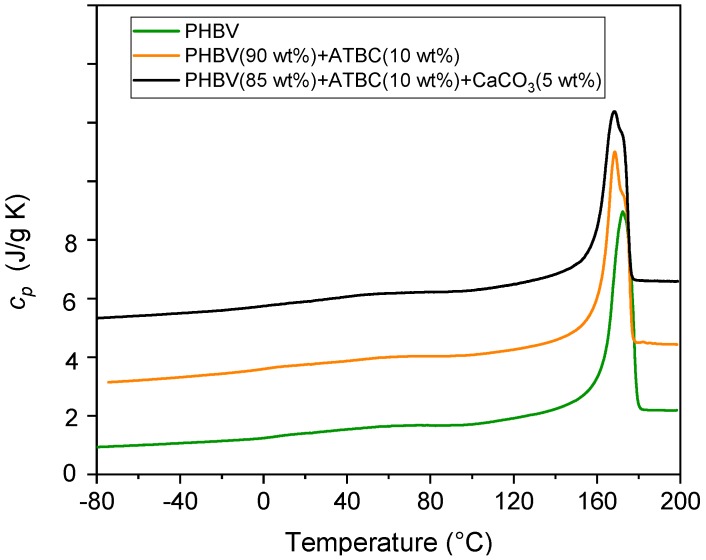
Specific heat capacity (*c_p_*) of PHBV and PHBV mixed with (*i*) ATBC and (*ii*) ATBC and the mineral filler CaCO_3_ at the concentrations indicated. The curves were obtained upon heating at 10 K/min after previous fast cooling to −85 °C. The ordinate values refer only to the bottom curve. All the other curves are shifted vertically for the sake of clearness.

The specific heat capacity (*c_p_*) curves of PHBV, and PHBV mixed with (*i*) the plasticizer ATBC, and (*ii*) with ATBC and the mineral filler CaCO_3_, measured at 10 K/min, are shown in Figure 3. As described in the section Materials and Methods, the samples were processed for 1 min at 80 °C. 

The glass transition of PHBV, which occurs in proximity of 5 °C, in agreement with literature data [42], is scarcely visible due to the high crystallinity of the samples. For this reason, no appreciable variation of *T_g_* in the presence of (*i*) ATBC and (*ii*) ATBC and the mineral filler CaCO_3_ can been detected. The glass transition is followed by an endothermic peak, centered around 60 °C, which was connected to initial partial melting and enthalpy recovery of the rigid amorphous fraction [43]. The main melting extends from approximately 120 °C to 180 °C, and shifts to slightly lower temperatures in the PHBV samples mixed with the plasticizer ATBC and the mineral filler CaCO_3_, due to a lower perfection of the PHBV crystal in the presence of additives [44]. A multiple melting behavior is exhibited by PHBV after addition of ATBC, which proves that reorganization and recrystallization occur at higher extent in the presence of the plasticizer, due to the enhanced mobility of the PHBV chains. Reorganization and recrystallization events, which generally take place in semi-crystalline polymers at a relatively low heating rate [45,46], have also been widely discussed and rationalized for the homopolymer PHB [47]. 

Table 3 lists the measured enthalpy of fusion (Δ*h_m_*), normalized to the PHBV content, and the crystalline weight fraction (*w_C_*) calculated from the Δ*h_m_* values divided by the enthalpy of fusion of 100% crystalline PHBV, assumed equal to that of the homopolymer PHB (Δ*h_m_*° = 143 J/g) [47]. The *w_C_* value are found to increase in the presence of ATBC and further after addition of CaCO_3_, which means that (*i*) the plasticizer, by enhancing the chain mobility, favors the crystal growth in PHBV, and that (*ii*) the mineral filler acts as nucleating agent for PHBV.

**Figure 4:**
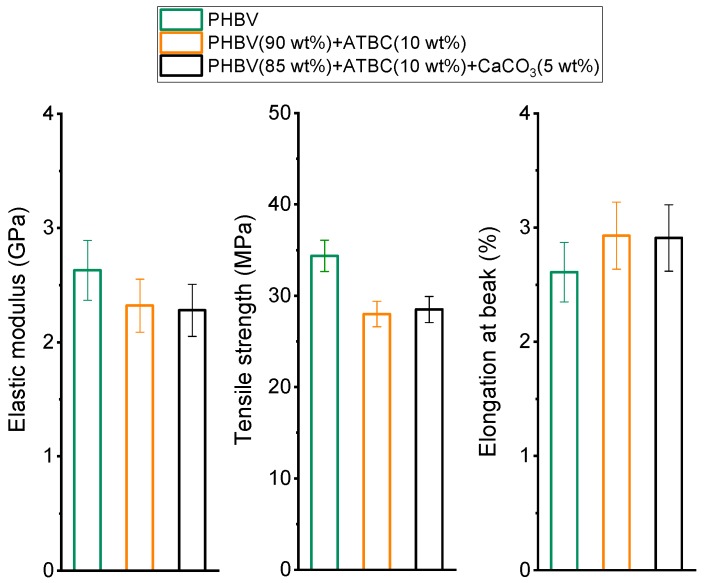
Elastic modulus, tensile strength and elongation at break of PHBV and PHBV mixed with (*i*) ATBC and (*ii*) ATBC and the mineral filler CaCO_3_ at the concentrations indicated (estimated errors from standard deviation for elastic modulus, and elongation at break: ± 10%; for tensile strength ± 5%).

The mechanical properties of PHBV and PHBV after processing in the presence of (*i*) the plasticizer ATBC, and (*ii*) the plasticizer ATBC and the mineral filler CaCO_3_ are summarized in Figure 4. PHBV is a brittle polymer, with a high elastic modulus and tensile strength. The addition of the plasticizer ATBC, as expected, modifies slightly the mechanical properties: the elastic modulus and the tensile strength decrease, and, conversely, the elongation at break increases. Despite the slightly higher crystallinity, which generally leads to an increase in the elastic modulus and in the tensile strength, and a decrease in the elongation at break, in the presence of the plasticizer ATBC the intermolecular forces between the PHBV chains become weaker, the mobility of the polymeric chains enhances, and a decrease in strength and an increase in flexibility and ductility is produced. Similar behaviors were reported for other PHBV plasticized systems [31,48,49]. The addition of the mineral filler CaCO_3_ has negligible further effect on the mechanical properties of PHBV. Although mineral particles generally act as stress concentrators, capable of initiating cracking and favoring specific and/or different fracture mechanisms, the influence of these fillers on the mechanical properties of a polymer can be small if present in low percentage. In this case, it can be assumed that the ‘perturbed’ polymer fraction around each mineral particle is low compared with the “unperturbed” one.

### 3.4. Thermal and Mechanical Properties of the PHBV Based Biocomposites without and with Surface Treatment of the Potato Pulp Power with Waxes

The thermal and mechanical properties of the biocomposite of PHBV with 20 wt % of PP powder were investigated in order to quantify how the addition of these fibers modifies the structure of the polymeric material. These data together provide information on possible miscibility/compatibility between the PHBV and the potato pulp.

In an attempt to improve compatibility between PHBV and the potato pulp powder, some bio-based and petroleum-based waxes were used as compatibilizers, through a fiber coating treatment. The thermal and mechanical properties of the PHBV based biocomposites in the presence of compatibilizers were also studied. 

**Figure 5:**
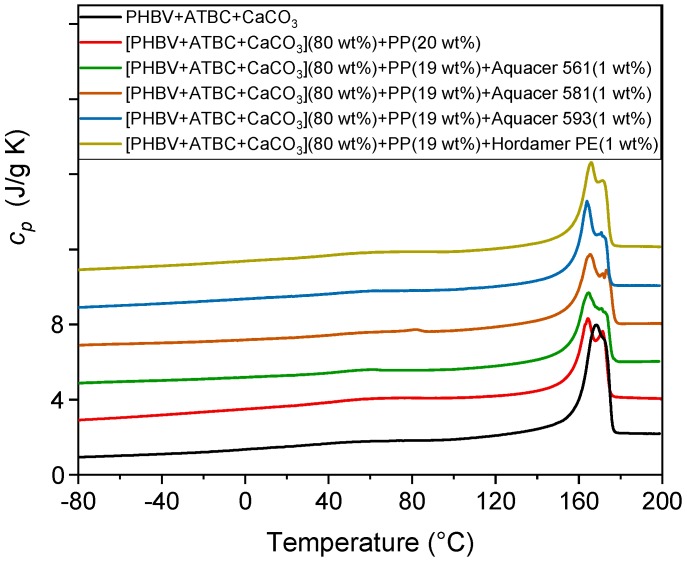
Specific heat capacity (*c_p_*) of the PHBV matrix and biocomposites indicated in the legend. The curves were obtained upon heating at 10 K/min after previous fast cooling to −85 °C. The ordinate values refer only to the bottom curve. All the other curves are shifted vertically for the sake of clearness.

The specific heat capacity (*c_p_*) curves of the PHBV based matrix and biocomposites with PP powder non-treated and treated with the waxes, measured at 10 K/min, are shown in Figure 5. As described in the Materials and Methods section, all the samples were processed for 1 min at 80 °C. The main melting process of the waxes, which is not visible in the *c_p_* curves due to their small amount, is located at approximately 65 °C, 85 °C, 160 °C and 95 °C, for Aquacer 561, Aquacer 581, Aquacer 593, and Hordamer PE, respectively.

Figure 5 shows that a multiple melting behavior is exhibited by the all the samples, which attests to the occurring of reorganization and recrystallization processes upon heating. However, for the PHBV based biocomposites the melting temperature of the first peak shifts to slightly lower temperatures, due to a lower perfection of the PHBV crystals that grow in the presence of the potato pulp powder.

Table 4 lists the measured enthalpy of fusion (Δ*h_m_*), measured from the *c_p_* curves plotted in Figure 5, after normalization to the PHBV amount, and the crystalline weight fraction (*w_C_*) calculated from the Δ*h_m_* values divided by the enthalpy of fusion of 100% crystalline PHBV (Δ*h_m_*° = 143 J/g) [47]. The *w_C_* value of the biocomposites containing fibers treated with the waxes are, within the experimental error, very close to those of the PHBV matrix and formulations without waxes. This attests that the PP powder, also after surface treatment with waxes, does not act as nucleating agent for the crystallization of PHBV. 

**Figure 6:**
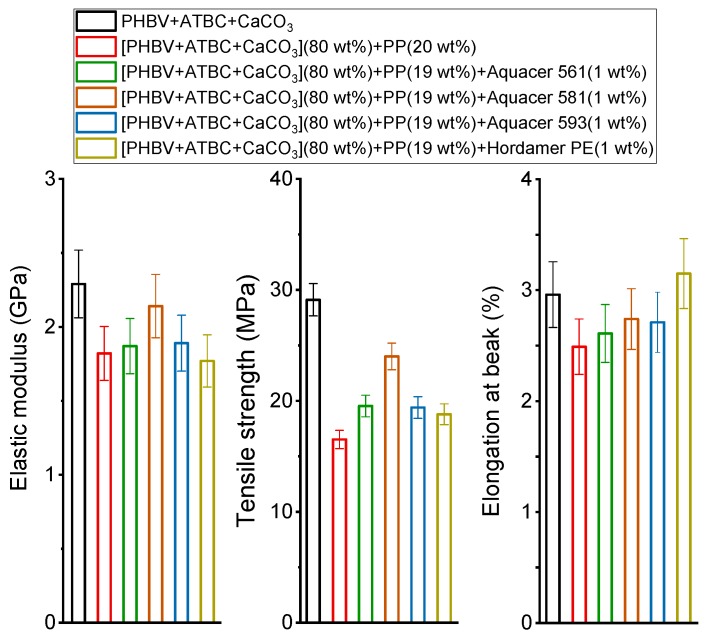
Elastic modulus, tensile strength and elongation at break of the PHBV based biocomposites indicated in the legend (estimated errors from standard deviation for elastic modulus, and elongation at break: ± 10%; for tensile strength ± 5%).

The mechanical properties of the PHBV based matrix and biocomposites with PP powder non-treated and treated with the bio-based and petroleum-based waxes are summarized in Figure 6. The elastic modulus of the biocomposite containing non-treated PP is found to be smaller with respect to the PHBV matrix, as well as the tensile strength and the elongation at break. The loss in the mechanical properties cannot be ascribed to PHBV degradation in the presence of potato pulp, which is supposed not to occur during processing at 180 °C for a short time, as discussed above. This assumption is confirmed by a study that proved that the molar mass of the PHBV matrix and the final mechanical properties of biocomposites with lignocellulosic fibers are negligibly affected by processing at about 180 °C, also in the presence of a small amount of moisture [50]. The worsening of the mechanical properties can be ascribed to the low aspect ratio of the potato pulp particles, so that PP powder acts for PHBV as filler, and not as reinforcement. Poor adhesion between the PP powder and the polymeric matrix is attested also by the lower tensile strength exhibited by the biocomposite. In regards to the elongation at break of the biocomposite, it appears to be smaller than that of the polymeric matrix because the dispersed filler particles act as stress concentrators. The incorporation of the PP powder to PHBV inhibits the deformation, which leads to reduced ductility of the material. Poor interactions are common in PHBV/lignocellulosic composites [39,51,52] and in PHBV/starch blends [53], because lignocellulosic fillers and starch are strongly hydrophilic whereas the PHBV is more hydrophobic.

Figure 6 also reveals that the surface treatment of the fibers with waxes can improve the mechanical properties. The best results are obtained by using the natural bio-based wax Aquacer 581. The elastic modulus, the tensile strength and the elongation at break of the biocomposite with PP powder treated with the carnauba wax (Aquacer 581) increase with respect to biocomposites with non-treated PP. This proves that the matrix/fiber adhesion improves and that better interfacial interactions are established between the polymer matrix and the potato pulp particles, which are made more hydrophobic by the incorporation of the carnauba wax. The increased interaction results in an improved load and stress transfer between the polymeric matrix and the filler. 

The treatment of PP powder with the bio-based wax Aquacer 561 (bee wax) does not cause significant changes in the elastic modulus and elongation at break of the biocomposite, although a higher tensile strength proves a better adhesion between the polymeric matrix and the filler. Similar effects on the mechanical properties of the PHBV biocomposite are provided by the surface treatment of PP powder with the petroleum-based waxes.

### 3.5. Morphology of the PHBV Based Biocomposites without and with Surface Treatment of the Potato Pulp Power with Waxes

**Figure 7:**
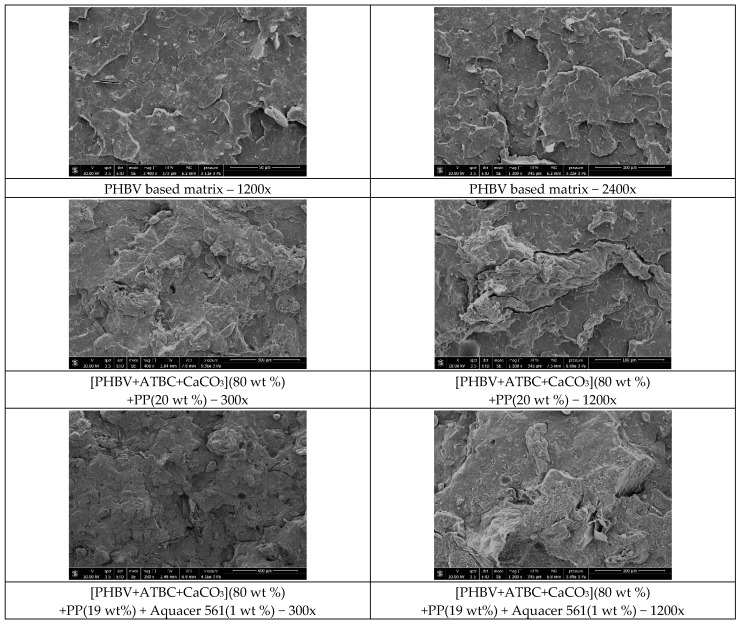

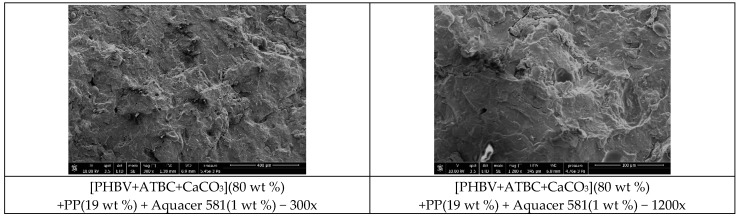
SEM images of the PHBV based matrix and biocomposites at the indicated magnification.

A morphological characterization by SEM was performed on fragments of dog-bone specimens of the PHBV matrix and biocomposites with PP powder non-coated and coated with natural waxes, with the aim of investigating the dispersion of the PP particles and the adhesion between the polymer matrix and the filler. Figure 7 shows that the topology of the pure PHBV matrix appears quite smooth and without evident voids. In the PHBV based biocomposite containing 20 wt % of PP powder, the filler particles appear well dispersed within the matrix and their distribution uniform, which means that they were satisfactorily separated during the extrusion process. The micrograph at 1200x magnification of the biocomposite [PHBV(85 wt %)+ATBC(10 wt %)+CaCO_3_(5 wt %)](80 wt %)+PP(20 wt %) clearly shows that the interfacial adhesion between the PHBV matrix and the PP power is quite poor, because the fiber appears pulled out. This explains the decrease in the tensile strength that is observed after addition of the PP powder to the PHBV matrix (Figure 6).

SEM observations on the PHBV based biocomposite after filler surface treatment with bee and carnauba waxes (Aquacer 561 and Aquacer 581, respectively) indicate substantial differences with respect to the untreated biocomposites. After filler coating, the fracture surface of the PHBV based biocomposite appears to be locally continuous and smoother, indicating that the compatibility of the matrix and the fibers is improved, which leads to better mechanical tensile properties, as previously discussed (Figure 6). Thus the surface treatment of the PP powder with the natural bee and carnauba waxes is proven to improve the wetting of the PHBV matrix, which results in a better PHBV/PP powder adhesion. The better adhesion between PHBV and the potato pulp particles can be ascribed to the composition of the bio-based Aquacer 561 and Aquacer 581 waxes. Both carnauba and bee waxes are rich in esters and hydroxyl esters (approximately 50% and 15% in constituent composition, respectively) [54,55], which can favor interactions with both the polymeric matrix and the hydrophilic potato pulp powder, made mainly of lignocellulosic fibers and starch.

## 4. Conclusions

The PP power utilized to produce biocomposites acts as filler, and not as reinforcement, for the PHBV matrix. The loss in mechanical properties is however not very high: the elastic modulus decreases by about 20% when the PP powder concentration is 20 wt %, with the result that this biocomposite still presents properties valuable for practical applications. The adhesion between the PP powder and the polymeric matrices is poor, as attested by the tensile strength, which has been found to be smaller for the biocomposite with respect to the PHBV matrix. The decrease is of about 40% when the PP powder concentration is 20 wt %. Also the ductility of the biocomposites is slightly smaller with respect to that of the PHBV polymeric matrix, because the incorporation of the PP powder promotes microcracks formation at the polymer/filler interface. 

An interesting result obtained by the present study is that a simple surface coating of the PP power with a bio-based wax, carnauba wax, can markedly improve the mechanical properties of the PHBV based biocomposite. Adhesion between the polymeric matrix and the filler improves also when another natural wax, bee wax, is used as surface coating of the PP powder. The use of bio-based waxes does not hinder biodegradability: on the contrary it allows to maintain a high content of bio-based components in the biocomposites.

In conclusion PP powder, which is an organic waste from the production and extraction of starch and no-food competition biomass, can be suitable for processing in the melt with the bio-based and biodegradable PHBV, presenting no main problems in processing. Due to the low aspect ratio of the PP powder, there is not a reinforcing effect on the polymeric matrix, but rather a moderate loss in mechanical properties that anyway still meet the technical requirements indicated for rigid packaging production. All the components of the biocomposites, including the natural waxes, are in the list of food contact approved substances, thus even food packaging application can be considered. The addition to PHBV of potato pulp powder up to about 20 wt % offers the possibility to reduce the cost of the final products, considering the relatively high cost of this polymer, and to make available the utilization and valorization of an abundant agro-food biomass such as potato pulp, according to the principles of the circular economy. 

## Figures and Tables

**Table 1:** Composition of the PHBV based matrix and biocomposites.

	Potato Pulp	Natural Wax
PHBV(100%)	-	-
PHBV(90 wt %)+ATBC(10 wt %)		
PHBV(85 wt %)+ATBC(10 wt %)+CaCO_3_(5 wt %)		
[PHBV(85 wt %)+ATBC(10 wt %)+CaCO_3_(5 wt %)](80 wt %)	PP(20 wt %)	
[PHBV(85 wt %)+ATBC(10 wt %)+CaCO_3_(5 wt %)](80 wt %)	PP(19 wt %)	Aquacer 561 (1 wt %)
[PHBV(85 wt %)+ATBC(10 wt %)+CaCO_3_(5 wt %)](80 wt %)	PP(19 wt %)	Aquacer 581 (1 wt %)
[PHBV(85 wt %)+ATBC(10 wt %)+CaCO_3_(5 wt %)](80 wt %)	PP(19 wt %)	Aquacer 593 (1 wt %)
[PHBV(85 wt %)+ATBC(10 wt %)+CaCO_3_(5 wt %)](80 wt %)	PP(19 wt %)	Hordamer PE (1 wt %)

**Table 2:** Operating condition use for the extrusion and injection molding process.

Extrusion Temperature (°C)	Screw Speed (rpm)	Cycle Time (s)	Injection Temperature (°C)	Injection Pressure (bar)	Molding Time (s)	Mold Temperature (°C)
180	100	90	180	150	60	80

**Table 3:** Enthalpy of fusion (Δ*h_m_*), and crystalline weight fraction (*w_C_*) for PHBV and PHBV mixed with (*i*) ATBC and (*ii*) ATBC and the mineral filler CaCO_3_ (estimated errors: ± 1 J/g for Δ*h_m_*, and ± 0.02 for *w_C_*).

	Δ*h_m_* (J/g)	*w_C_*
PHBV(100%)	92	0.65
PHBV(90 wt %)+ATBC(10 wt %)	97	0.68
PHBV(85 wt %)+ATBC(10 wt %)+CaCO_3_(5 wt %)	103	0.72

**Table 4:** Enthalpy of melting (Δ*h_m_*), and crystalline weight fraction (*w_C_*) of PHBV based matrix and biocomposites (estimated errors: ± 1 J/g for Δ*h_m_*, and ± 0.02 for *w_C_*).

	Δ*h_m_* (J/g)	*w_C_*
PHBV(85 wt %)+ATBC(10 wt %)+CaCO_3_(5 wt %)	103	0.72
[PHBV(85 wt %)+ATBC(10 wt %)+CaCO_3_(5 wt %)](80wt %)+PP(20 wt %)	103	0.72
[PHBV(85 wt %)+ATBC(10 wt %)+CaCO_3_(5 wt %)](80 wt %)+PP(19 wt %)+Aquacer 561 (1 wt %)	103	0.68
[PHBV(85 wt %)+ATBC(10 wt %)+CaCO_3_(5 wt %)](80 wt %)+PP(19 wt %)+Aquacer 581 (1 wt %)	101	0.71
[PHBV(85 wt %)+ATBC(10 wt %)+CaCO_3_(5 wt %)](80 wt %)+PP(19 wt %)+Aquacer 593 (1 wt %)	101	0.71
[PHBV(85 wt %)+ATBC(10 wt %)+CaCO_3_(5 wt %)](80 wt %)+PP(19 wt %)+Hordamer PE (1 wt %)	101	0.71

## References

[1] Padovani G., Carlozzi P., Seggiani M., Cinelli P., Vitolo S., Lazzeri A. (2016). PHB-rich biomass and BioH2 production by means of photosynthetic microorganisms. Chem. Eng. Trans..

[2] Bugnicourt E., Cinelli P., Lazzeri A., Alvarez V. (2014). Polyhydroxyalkanoate (PHA): Review of synthesis, Characteristics, processing and potential applications in packaging. eXPRESS Polym. Lett..

[3] Sudesh K., Abe H., Doi Y. (2000). Synthesis, structure and properties of polyhydroxyalkanoates: Biological polyesters. Prog. Polym. Sci..

[4] Laycock B., Halley P., Pratt S., Werker A., Lant P. (2013). The chemomechanical properties of microbial polyhydroxyalkanoates. Progr. Polym. Sci..

[5] Keskin G., Kızıl G., Bechelany M., Pochat-Bohatier C., Öner M. (2017). Potential of polyhydroxyalkanoate (PHA) polymers family as substitutes of petroleum based polymers for packaging applications and solutions brought by their composites to form barrier materials. Pure Appl. Chem..

[6] Deroiné M., Le Duigou A., Corre Y.M., Le Gac P.Y., Davies P., César G., Bruzaud S. (2014). Seawater accelerated ageing of poly(3-hydroxybutyrate-co-3-hydroxyvalerate). Polym. Degrad. Stab..

[7] Volova T.G., Boyandin A.N., Vasiliev A.D., Karpov V.A., Prudnikova S.V., Mishukova O.V., Boyarskikh U.A., Filipenko M.L., Rudnev V.P., Xuân B.B. (2010). Biodegradation of polyhydroxyalkanoates (PHAs) in tropical coastal waters and identification of PHA-degrading bacteria. Polym. Degrad. Stab..

[8] Seggiani M., Cinelli P., Balestri E., Mallegni N., Stefanelli E., Rossi A., Lardicci C., Lazzeri A. (2018). Novel Sustainable Composites Based on Poly(hydroxybutyrate-co-hydroxyvalerate) and Seagrass Beach-CAST Fibers: Performance and Degradability in Marine Environments. Materials.

[9] Misra M., Pandey J., Mohanty A. (2015). Biocomposites: Design and Mechanical Performance.

[10] Dipa R. (2017). Biocomposites for High-Performance Applications.

[11] Jawaid M., Thariq M., Sabe N. (2019). Mechanical and Physical Testing of Biocomposites, Fibre-Reinforced Composites and Hybrid Composites.

[12] Bledzki A.K., Gassan J. (1999). Composites reinforced with cellulose based fibers. Prog. Polym. Sci..

[13] Mohanty A.K., Misra M., Hinrichsen G. (2000). Biofibers, biodegradable polymers and biocomposites: An overview. Macromol. Mater. Eng..

[14] Yua L., Deana K., Li L. (2006). Polymer blends and composites from renewable resources. Prog. Polym. Sci..

[15] Maya-Jacob J., Sabu T. (2008). Review—Biofibres and biocomposites. Carbohydr. Polym..

[16] Satyanarayana K.G., Arizaga Guadalupe Carbajal G., Wypych F. (2009). Polymer blends and composites from renewable resources Biodegradable composites based on lignocellulosic fibers—An overview. Prog. Polym. Sci..

[17] Faruka O., Bledzkia A.K., Fink H.P., Saind M. (2012). Biocomposites reinforced with natural fibers: 2000–2010. Prog. Polym. Sci..

[18] Pandey J.K., Ahn S.H., Lee C.S., Mohanty A.K., Misra M. (2010). Recent Advances in the Application of Natural Fiber Based Composites. Macromol. Mater. Eng..

[19] Nielsen L.E., Landel R.F. (1994). Mechanical Properties of Polymers and Composites.

[20] Ku H., Wang H., Pattarachaiyakoop N., Trada M. (2011). A review on the tensile properties of natural fiber reinforced polymer composites. Composites B.

[21] Signori F., Pelagaggi M., Bronco S., Righetti M.C. (2012). Amorphous/Crystal and Polymer/Filler Interphases in Biocomposites from Poly(butylene succinate). Thermochim. Acta.

[22] Jiang L., Huang J., Qian J., Chen F., Zhang J., Wolcott M.P., Zhu Y. (2008). Study of Poly(3-hydroxybutyrate-co-3-hydroxyvalerate) (PHBV)/Bamboo Pulp Fiber Composites: Effects of Nucleation Agent and Compatibilizer. J. Polym. Environ..

[23] Shanks R.A., Hodzic A., Wong S. (2004). Thermoplastic biopolyester natural fiber composites. J. Appl. Polym. Sci..

[24] Keller A. (2003). Compounding and mechanical properties of biodegradable hemp, fiber composites. Compos. Sci. Techol..

[25] Muthuraj R., Misra M., Mohanty A.K. (2017). Reactive compatibilization and performance evaluation of miscanthus biofiber reinforced poly(hydroxybutyrate-co-hydroxyvalerate) biocomposites. J. Appl. Polym. Sci..

[26] Montano-Leyva B., Gontard N., Angellier-Coussy H. (2017). Poly(3-hydroxybutyrate-co-hydroxyvalerate) and wheat straw fibers biocomposites produced by co-grinding: Processing and mechanical behavior. J. Compos. Mater..

[27] Srubar W.V., Pilla S., Wright Z.C., Ryan C.A., Greene J.P., Frank C.W., Billington S.L. (2012). Mechanisms and impact of fiber–matrix compatibilization techniques on the material characterization of PHBV/oak wood flour engineered biobased composites. Compos. Sci. Technol..

[28] Väisänen T., Haapala A., Lappalainen R., Tomppo L. (2016). Utilization of agricultural and forest industry waste and residues in natural fiber-polymer composites: A review. Waste Manag..

[29] Fritsch C., Staebler A., Happel A., Cubero Márquez M.A., Aguiló-Aguayo I., Abadias M., Gallur M., Cigognini I.M., Montanari A., López M.J. (2017). Processing, Valorization and Application of Bio-Waste Derived Compounds from Potato, Tomato, Olive and Cereals: A Review. Sustainability.

[30] Schmid M., Herbst C., Mueller K., Staebler A., Schlemmer D., Coltelli M.B., Lazzeri A. (2016). Effect of potato pulp filler on the mechanical properties and water vapor transmission rate of thermoplastic WPI/PBS blends. Polym. Plast. Technol. Eng..

[31] Wang L., Zhu W., Wang X., Chen X., Chen G.Q., Xu K. (2008). Processability Modifications of Poly(3-hydroxybutyrate) by Plasticizing, Blending, and Stabilizing. J. Appl. Polym. Sci..

[32] Quintana R., Persenaire O., Lemmouchi Y., Sampson J., Martin S., Bonnaud L., Dubois P. (2013). Enhancement of cellulose acetate degradation under accelerated weathering by plasticization with eco-friendly plasticizers. Polym. Degrad. Stab..

[33] European Food Safety Authority (2012). Scientific Opinion on Flavouring Group Evaluation 10, Revision 3 (FGE.10Rev3): Aliphatic primary and secondary saturated and unsaturated alcohols, aldehydes, acetals, carboxylic acids and esters containing an additional oxygenated functional group and lactones from chemical groups 9, 13 and 30. EFSA J..

[34] Sarge S.M., Hemminger W., Gmelin E., Höhne G.W.H., Cammenga H.K., Eysel W. (1997). Metrologically based procedures for the temperature, heat and heat flow rate calibration of DSC. J. Therm. Anal..

[35] Yang H., Yan R., Chen H., Lee D.H., Zheng C. (2007). Characteristic of hemicellulose, cellulose and lignin pyrolysis. Fuel.

[36] Garcia-Perez M., Chaala A., Yang J., Roy C. (2001). Co-pyrolysis of sugarcane bagasse with petroleum residue. Part I: Thermogravimetric analysis. Fuel.

[37] Aggarwal P., Dollimore D., Heon K. (1997). Comparative thermal analysis study of two biopolymers, starch and cellulose. J. Therm. Anal..

[38] Kamur P., Sandeep K.P., Alavi S., Truong V.D., Gorga R.E. (2010). Preparation and characterization of bio-nanocomposite films based on soy protein isolate and montmorillonite using melt extrusion. J. Food Eng..

[39] Singh S., Mohanty A.K., Sugie T., Takai Y., Hamada H. (2008). Renewable resource based biocomposites from natural fiber and polyhydroxybutyrate-co-valerate (PHBV) bioplastics. Composites Part A.

[40] Srithep Y., Ellingham T., Peng J., Sabo R., Clemons C., Turng L.-S., Pilla S. (2013). Melt compounding of poly(3-hydroxybutyate-co-3-hydroxyvalerate)/nanofibrilled cellulose nanocomposites. Polym. Degrad. Stab..

[41] Batista K.C., Silva D.A.K., Coelho L.A.F., Pezzin S.H., Pezzin A.P.T. (2010). Soil Biodegradation of PHBV/Peach Palm Particles Biocomposites. J. Polym. Environ..

[42] Czerniecka A., Magon A., Schliesser J., Woodfield B.F., Pyda M. (2014). Heat capacity of poly(3-hydroxybutyrate). J. Chem. Thermodyn..

[43] Di Lorenzo M.L., Gazzano M., Righetti M.C. (2012). The role of the rigid amorphous fraction on cold crystallization of poly(3-hydroxybutyrate). Macromolecules.

[44] Yoshie N., Nakasato K., Fujiwara M., Kasuya K., Abe H., Doi Y., Inoue Y. (2000). Effect of low molecular weight additives on enzymatic degradation of poly(3-hydroxybutyrate). Polymer.

[45] Minakov A.A., Mordvintsen D.A., Schick C. (2004). Melting and reorganization of poly(ethylene terephthalate) on fast heating (1000 K/s). Polymer.

[46] Righetti M.C., Laus M., Di Lorenzo M.L. (2014). Temperature dependence of the rigid amorphous fraction in poly(ethylene terephthalate). Eur. Polym. J..

[47] Righetti M.C., Tombari E., Di Lorenzo M.L. (2013). The role of the crystallization temperature on the nanophase structure evolution of poly([(R)-3-hydroxybutyrate). J. Phys. Chem. B.

[48] Baltieri R.C., Innocentini Mei L.H., Bartoli J. (2003). Study of the influence of plasticizers on the thermal and mechanical properties of poly(3-hudroxybutyrate) compounds. Macromol. Symp..

[49] Kurusu R.S., Siliki C.A., David E., Demarquette N.R., Gauthier C., Chenal J.M. (2015). Incorporation of plasticizers in sugarcane-based poly(3-hydroxybutyrate)(PHB): Changes in microstructure and properties through ageing and annealing. Ind. Crop. Prod..

[50] Berthet M.-A., Gontard N., Angellier-Coussy H. (2015). Impact of fiber moisture content on the structural/mechanical properties relationships of PHBV/wheat straw fibers biocomposites. Composites A.

[51] Barkoula N.M., Garkhail S.K., Peijs T. (2010). Biodegradable composites based on flax/polyhydroxybutyrate, and its copolymer with hydroxyvalerate. Ind. Crops Prod..

[52] Berthet M.-A., Angellier-Coussy H., Chea V., Guillard V., Gastaldi E., Gontard N. (2015). Sustainable food packaging: Valorizing wheat straw fibers for tuning PHBV-based composites properties. Compos. Sci. Technol..

[53] Reis K.C., Pereira J., Smith A.C., Carvalho C.W.P., Wellner N., Yakimets I. (2008). Characterization of polyhydroxybutyartehydroxyvalerate (PHB-HV)/maize starch blend films. J. Food Eng..

[54] Vandenburg L.E., Wilder E.A. (1970). The Structural Constituents of Carnauba Wax. J. Am. Oil Chem. Soc..

[55] Tulloch A.P. (1970). The Composition of Beeswax and Other Waxes Secreted by Insect. Lipids.

